# Bv8 Blockade Sensitizes Anti-PD1 Therapy Resistant Tumors

**DOI:** 10.3389/fimmu.2022.903591

**Published:** 2022-07-07

**Authors:** Madeleine Benguigui, Avital Vorontsova, Michael Timaner, Sapir Levin, Jozafina Haj-Shomaly, Abhilash Deo, Rotem Menachem, Bar Manobla, Tim J. Cooper, Ziv Raviv, Yuval Shaked

**Affiliations:** ^1^ Department of Cell Biology and Cancer Science, Rappaport Faculty of Medicine, Technion – Israel Institute of Technology, Haifa, Israel; ^2^ Rappaport Technion Integrated Cancer Center Technion - Israel Institute of Technology, Haifa, Israel; ^3^ Faculty of Chemical engineering, Technion- Israel Institute of Technology, Haifa, Israel; ^4^ Department of Immunology, Rappaport Faculty of Medicine, Technion – Israel Institute of Technology, Haifa, Israel

**Keywords:** immunotherapy, MDSC, resistance, Bv8, tumor microenvironment

## Abstract

Myeloid-derived suppressor cells (MDSCs) are known to promote tumor growth in part by their immunosuppressive activities and their angiogenesis support. It has been shown that Bv8 blockade inhibits the recruitment of MDSCs to tumors, thereby delaying tumor relapse associated with resistance to antiangiogenic therapy. However, the impact of Bv8 blockade on tumors resistant to the new immunotherapy drugs based on the blockade of immune checkpoints has not been investigated. Here, we demonstrate that granulocytic-MDSCs (G-MDSCs) are enriched in anti-PD1 resistant tumors. Importantly, resistance to anti-PD1 monotherapy is reversed upon switching to a combined regimen comprised of anti-Bv8 and anti-PD1 antibodies. This effect is associated with a decreased level of G-MDSCs and enrichment of active cytotoxic T cells in tumors. The blockade of anti-Bv8 has shown efficacy also in hyperprogressive phenotype of anti-PD1-treated tumors. *In vitro*, anti-Bv8 antibodies directly inhibit MDSC-mediated immunosuppression, as evidenced by enhanced tumor cell killing activity of cytotoxic T cells. Lastly, we show that anti-Bv8-treated MDSCs secrete proteins associated with effector immune cell function and T cell activity. Overall, we demonstrate that Bv8 blockade inhibits the immunosuppressive function of MDSCs, thereby enhancing anti-tumor activity of cytotoxic T cells and sensitizing anti-PD1 resistant tumors. Our findings suggest that combining Bv8 blockade with anti-PD1 therapy can be used as a strategy for overcoming therapy resistance.

## Introduction

Cancer immunotherapy has undergone a quantum leap in recent years owing to the elucidation of mechanisms underlying tumor immune escape. The discovery of immune checkpoint proteins has led to the development of a new generation of cancer immunotherapies in the form of immune checkpoint inhibitors (ICIs) that activate anti-tumor immunity mediated by host cells (1). CTLA-4, PD-1 and its ligand, PD-L1 are immune checkpoint proteins for which therapeutic antibodies have been developed and approved by the FDA for the treatment of a variety of cancers (2). These drugs demonstrate promising and remarkable successes for the treatment of advanced malignancies such as melanoma, non-small cell lung cancer (NSCLC), renal cell carcinoma, and some hematological malignancies (2–6). However, the therapeutic benefit of these drugs is limited to a small proportion of treated patients, with the majority of patients considered intrinsically resistant to such therapies (7), some of whom may even display a hyperprogressive disease (8, 9). Thus, additional preclinical and clinical research focusing on the potential mechanisms of resistance to ICI therapy is worthy.

Myeloid-derived suppressor cells (MDSCs) are a heterogeneous population of immunosuppressive, immature myeloid cells implicated in various pathological conditions (10). MDSCs are classically subdivided into two major categories: neutrophil-like, polymorphonuclear MDSCs (PMN-MDSCs, defined as CD11b^+^ Ly6G^hi^)), and monocyte-like, monocytic MDSCs (M-MDSCs, defined as CD11b^+^ Ly6C^hi^) (11) (12, 13). The relative abundance of MDSCs in the tumor microenvironment is associated with clinical outcome and responsiveness to anticancer drugs (14). Previous studies highlighted the pro-angiogenic activity of MDSCs in tumors. Increased levels of MDSCs contributed to tumor refractoriness in tumors treated with anti-VEGF antibodies (15). Among the factors secreted by MDSCs are prokineticin (Bv8), a protein supporting VEGF-independent tumor angiogenesis. Consequently, blocking the Bv8 axis decreases tumor expansion and recruitment of MDSCs leading to decreased tumor progression following antiangiogenic therapy resistance (16). Thus, anti-Bv8 antibodies have been developed as a drug to increase the duration of response of anti-VEGF therapy and minimize refractoriness, *via* the inhibition of MDSC recruitment to tumors.

In addition to their pro-angiogenic effect, MDSCs in tumors play a role in resistance to ICI therapy, owing to their immunosuppressive activity thereby inhibiting anti-tumor immunity (17). In this regard, we have previously demonstrated that the host effect to anti-tumor immunity derived by ICI therapy, is counteracted by elevated levels of immunosuppressive immune cells such as MDSCs and immunosuppressive macrophages (18). We demonstrated that these effects contributed to tumor regrowth negating the anti-tumor activity of the drug. Thus, these collective effects may contribute to immunotherapy resistance (19). However, the effect of blocking the recruitment of MDSCs to tumors using anti-Bv8 antibodies has never been studied.

Here we show that anti-Bv8 treatment sensitizes tumors otherwise resistant to anti-PD1 therapy in various preclinical models. We demonstrate that the addition of anti-Bv8 antibodies to anti-PD1 therapy increases cytotoxic T cell activity and reduces G-MDSCs in tumors. *In vitro*, anti-Bv8 antibodies inhibit the immunosuppressive function of MDSCs, and promote the secretion of factors involved in anti-tumor immunity. This study suggests that anti-Bv8 antibodies have the potential to be used therapeutically to sensitize ICI therapy-resistant tumors.

## Results

### Myeloid Cells Are Enriched in Anti-PD1 Resistant Tumors

**Figure 1:**
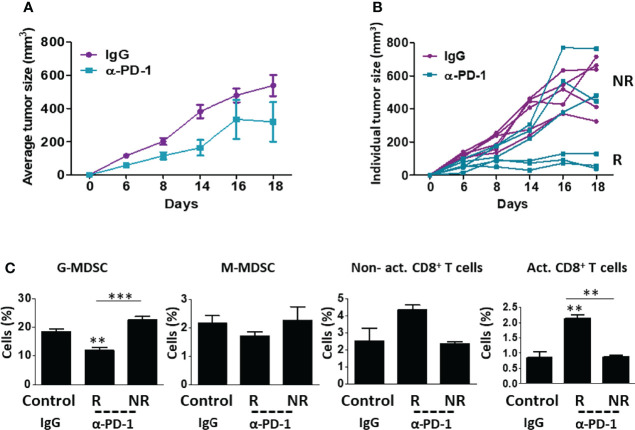
EMT6 tumors exhibit differential response to anti-PD1 therapy. **(A, B)** Eight-to-ten week old BALB/c mice (n=5-6/group) were implanted with EMT6 cells (5x10^5^/mouse) in the mammary fat pad. When tumors reached 50 mm^3^, treatment with anti-PD1 or IgG control antibodies was initiated twice a week for two weeks. Tumor growth was assessed regularly **(A)**. At end point, mice were stratified into groups based on their response to treatment. Responders (R) and non-responders (NR) are shown in the spider plot **(B)**. **(C)** Tumors from control mice (IgG) and anti-PD1-treated responder (R) and non-responder (NR) mice were removed and prepared as single-cell suspensions. Granulocytic and monocytic MDSCs (G-MDSC and M-MDSC, respectively) as well as non-activated and activated CD8+ T cells were quantified using flow cytometry and presented as the percentage from CD45+ cells. The average percentage ± SD for each cell type is shown in a bar graph. Statistical significance was assessed by one-way ANOVA followed by Tukey *post-hoc* test. Significant p values are shown as **p<0.01 and ***p<0.001 from control or otherwise indicated in the figure.

To identify immune cell types involved in ICI therapy resistance, we compared immune cell composition in orthotopically implanted breast carcinoma tumors displaying spontaneous sensitivity or resistance to treatment. To this end, BALB/c mice were implanted with EMT6 breast carcinoma cells which are known to partially respond to ICI therapy (18, 20). When tumors reached a size of 50 mm^3^, treatment with anti-PD1 or IgG control antibodies was initiated twice a week for two weeks. Tumor growth was assessed regularly. As expected, response to anti-PD1 treatment was observed in some, but not all, mice. Tumors that did not respond to treatment exhibited a similar growth rate to tumors implanted in control IgG-treated mice (**Figures 1A, B**). At day 18, a clear separation between responding and non-responding tumors to anti-PD1 therapy was observed, which indicated the endpoint of this experiment. At this point, mice were sacrificed, and tumors were prepared as single-cell suspensions to evaluate immune cell composition. Tumors that responded to anti-PD1 treatment exhibited a substantial decrease in G-MDSCs and an increase in lymphoid cells (mostly CD8+ T cells) in comparison to non-responding anti-PD1-treated tumors (**Figure 1C**, and **Figure S1A** for validation). Notably, no significant changes in the levels of immune cells were observed in the blood, although a trend towards decreased anti-tumor lymphoid cells was apparent in non-responding mice (**Figure S1B**). These results support previously published studies reporting the role of MDSCs in ICI therapy resistance (21).

### Bv8 Blockade Sensitizes Anti-PD1 Resistant Tumors

**Figure 2:**
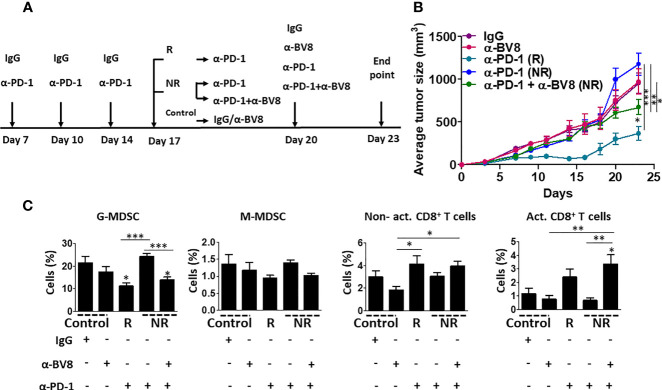
Bv8 blockade sensitizes anti-PD1 resistant tumors. Eight-to-ten week old BALB/c mice (n=6-10 mice/group) were implanted with EMT6 cells (5x10^5^/mouse) in the mammary fat pad. When tumors reached 50 mm^3^, treatment with anti-PD1 or IgG control antibodies was initiated twice weekly and tumor growth was monitored. After three drug administrations, the mice were stratified according to their response to treatment. Responder (R) mice continued anti-PD1 monotherapy. Non-responder (NR) mice were either treated with a combination of anti-PD1 and anti-Bv8 antibodies or continued anti-PD1 monotherapy. IgG-treated control mice were either switched to anti-Bv8 antibody monotherapy or continued receiving IgG control antibodies. **(A)** Treatment regimens for each group are shown. **(B)** Tumor growth per group is shown. **(C)** At the endpoint, tumors were removed and subsequently prepared as single-cell suspensions for the analysis of granulocytic and monocytic MDSCs (G-MDSC and M-MDSC, respectively) as well as non-activated and activated CD8+ T cells using flow cytometry. The results are presented as the percentage from CD45+ cells. The average percentage ± SD for each cell type, is shown in a bar graph. Statistical significance was assessed by one-way ANOVA followed by Tukey *post-hoc* test. Significant p values are shown as *p<0.05; **p<0.01; ***p<0.001 from control or otherwise indicated in the figure.

MDSCs have been demonstrated to support tumor angiogenesis and immunosuppression (11). Previous studies reported that Bv8 blockade inhibits the colonization of MDSCs in tumors and promotes anti-angiogenic activity independent of VEGF (22). We, therefore, asked whether inhibiting the tumor recruitment of MDSCs using anti-Bv8 antibodies sensitizes anti-PD1 resistant tumors. To this end, EMT6 tumors were implanted in BALB/c mice, and when tumors reached 50 mm^3^, treatment with anti-PD1 or IgG control antibodies was initiated for 10 days. The anti-PD1-treated mice were then stratified into groups based on their response to treatment (as shown in **Figure 1**). Anti-PD1 sensitive mice continued anti-PD1 monotherapy, while anti-PD1 resistant mice were either treated with a combination of anti-PD1 and anti-Bv8 antibodies or continued anti-PD1 monotherapy for one week. IgG-treated control mice were either switched to anti-Bv8 antibody monotherapy or continued receiving IgG control antibodies (**Figure 2A**). As shown in **Figure 2B**, the growth rate of anti-PD1 resistant tumors in mice receiving anti-PD1 monotherapy was similar to that in control mice receiving IgG or anti-Bv8 monotherapies. However, in comparison to these three groups, tumor growth was significantly reduced in mice treated with the combination of anti-PD1 and anti-Bv8 antibodies, suggesting a sensitization of the tumor to anti-PD1 therapy. At endpoint, tumors were removed, and immune cell composition was analyzed. Tumors from mice treated with a combination of anti-PD1 and anti-Bv8 antibodies exhibited a significant reduction in the levels of G-MDSCs and a significant increase in the levels of activated CD8+ T cells in comparison to anti-PD1 resistant tumors from mice receiving anti-PD1 monotherapy. In addition, a trend toward increased anti-tumor lymphoid cells (i.e., CD8+ T cells) was also observed in tumors from mice treated with the combination therapy (**Figure 2C** and **Figure S2A** for validation). Consistent with the results shown in **Figure S1**, no significant changes were observed in the levels of myeloid and lymphoid cells in the peripheral blood of mice in the different treatment groups (**Figure S2B**). Comparable effects on tumor growth rates and immune cell composition were observed in Lewis lung carcinoma (LLC) and renal cell cancer (RENCA) models, both of which are considered non-responsive to anti-PD1 therapy (23, 24). Evidently, in both tumor models, tumor growth was substantially inhibited in mice treated with anti-PD1 and anti-Bv8 combination therapy (**Figures S3A** and **S4A**). In addition, the tumor levels of G-MDSCs were significantly reduced, and activated CD8+ T cells were significantly increased in the combination therapy groups, while no changes in peripheral blood lymphoid and myeloid subsets were observed (**Figures S3B, C** and **S4B, C**). Of note, in the LLC tumor model, anti-Bv8 monotherapy reduced tumor growth to some extent, probably due to its anti-angiogenic effects (25, 26). In addition, we found the mRNA levels of Bv8 and its receptor PRK2 are primarily expressed in MDSCs and not in T cells or EMT6, RENCA, and LLC cancer cells. These results further indicate that anti-Bv8 therapy most likely affects the activity of MDSCs (**Figure S5**). Taken together, these results demonstrate that Bv8 blockade sensitizes anti-PD1 resistant tumors, probably due to its effect on inhibiting the tumor colonization of MDSCs and increasing the anti-tumor immunity.

### Bv8 Blockade Counteracts Tumor Hyperprogression Following Anti-PD1 Therapy

**Figure 3:**
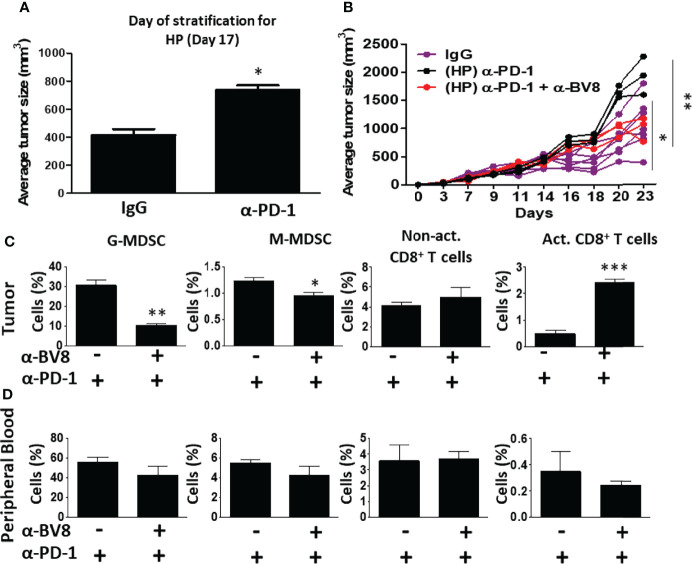
Anti-Bv8 treatment inhibits the growth of hyperprogressive tumors following anti-PD1 therapy. **(A)** A subset of EMT6 tumor-bearing BALB/c mice treated with anti-PD1 shown in **Figure 2**, displayed accelerated tumor growth, termed hyperprogression (HP) when compared to IgG control (n=6 mice), shown on day 17. At this time, the mice were treated with anti-PD1 in combination with anti-Bv8 or anti-PD1 as a monotherapy (n=3 mice/group). **(B)** Tumor growth in individual mice is shown in a spider plot. **(C, D)** At the endpoint, tumors and peripheral blood were harvested. Granulocytic and monocytic MDSCs (G-MDSC and M-MDSC, respectively) as well as non-activated and activated CD8+ T cells in tumor single-cell suspensions **(C)** and peripheral blood **(D)** were quantified by flow cytometry, and presented as the percentage from CD45+ cells. The average percentage ± SD for each cell type, is shown in a bar graph. Statistical significance was assessed using unpaired two-tailed t-test. Significant p values are shown as *p<0.05; **p<0.01; ***p<0.001.

A number of clinical studies demonstrate that ICI therapy is sometimes followed by tumor hyperprogression in some cancers (8, 27). For example, in head and neck squamous cell carcinoma, ~30% of ICI-treated patients display hyperprogressive tumors (28). In our EMT6 breast carcinoma model, a trend towards hyperprogressive tumor phenotype was observed in a subset of anti-PD1-treated mice already on day 17 following tumor implantation. Such mice displayed a faster tumor growth rate in comparison to IgG-treated control mice (**Figure 3A**). At this point (day 17), levels of G-MDSCs were significantly higher in the hyperprogressive tumors compared with responding (R) and non-responding (NR) tumors, while levels of activated cytotoxic T cells were substantially reduced when compared to responding (R) tumors. No significant changes in the levels of MDSCs and T cells were found in the blood (**Figure S6**). Interestingly, the addition of anti-Bv8 antibodies to anti-PD1 treatment on day 17, resulted in attenuated hyperprogression in comparison to mice treated with anti-PD1 monotherapy (**Figure 3B**). We also observed that tumors from the combined therapy group displayed a substantial decrease in G-MDSCs and an increase in activated cytotoxic T cells. Such changes were not observed in the blood (**Figures 3C, D**). These results indicate that Bv8 blockade counteracts the hyperprogressive tumor phenotype sometimes occurring following anti-PD1 therapy.

### Bv8 Blockade Enhances Cytotoxic T Cell Anti-Tumor Activity

**Figure 4:**
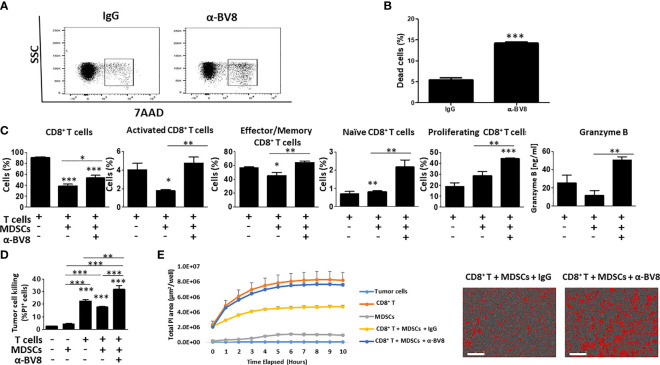
Anti-Bv8 treatment inhibits the immunosuppressive function of MDSCs. **(A, B)** MDSC (Gr1+ cells) were isolated from the spleens of EMT6 tumor-bearing mice. The cells were cultured with anti-Bv8 or IgG control antibodies for 24 hours. Cell viability was assessed by 7AAD using flow cytometry, and presented by dot plot **(A)** followed by a summary graph **(B)**. **(C)** MDSCs and CD8+ T cells were isolated from the spleen of EMT6 tumor-bearing mice. MDSCs and CD8+ T cells were co-cultured in the presence of anti-Bv8 or IgG control antibodies, and T cell state was evaluated by flow cytometry. Shown are percentages of CD8+ T cells, activated CD8+ T cells, Effector/memory CD8+ T cells, naïve CD8+ T cells, proliferating CD8+ T cells, and granzyme B expressed in the co-culture media. **(D, E)** MDSCs and CD8+ T cells isolated from the spleen of EMT6 tumor-bearing mice (n=5 mice) were cultured with EMT6 cells as described in Materials and Methods. Tumor cell killing was assessed by flow cytometry **(D)** and Incucyte **(E)**. Representative images at the 10-hour timepoint are shown on the right. Scale bar= 100μm. Statistical significance was assessed by one-way ANOVA followed by Tukey *post-hoc* test. Significant p values are shown as * p<0.05; ** p<0.01; *** p<0.001 from control or otherwise indicated in the figure.

To further understand how Bv8 blockade promotes the sensitization of anti-PD1 resistant tumors, we next studied the effect of anti-Bv8 antibodies on cytotoxic T cell activity *in vitro*. To this end, MDSCs and CD8+ T cells were isolated from splenocytes obtained from EMT6 tumor-bearing mice. Isolated MDSCs were first cultured with anti-Bv8 or IgG control antibodies and evaluated for apoptosis. An increase in the levels of apoptotic MDSCs was found in the presence of anti-Bv8 compared to control, further indicating that anti-Bv8 reduces MDSC viability (**Figures 4A, B**). We then further characterized the immunosuppressive activity of MDSCs in the presence of anti-Bv8 therapy. To this end, Gr1+ cells were isolated from the EMT6 tumors of mice treated with anti-Bv8 or control. The cells were then analyzed for several immune-modulating associated genes including IDO, ROS1, iNOS, and Arg1. The mRNA levels of IDO, ROS1, and iNOS were substantially higher in MDSCs obtained from anti-Bv8 treated tumors compared to control, while mRNA levels of Arg1 did not significantly change (**Figure S7**). These results further suggest that anti-Bv8 antibodies inhibit the immunosuppressive activity of MDSCs.

Next, MDSCs were co-cultured with CD8+ T cells in the presence or absence of anti-Bv8 antibodies, and the activity of CD8+ T cells was then assessed. As expected, MDSCs significantly reduced the percentage of activated and effector CD8+ T cells in comparison to the control culture comprised of CD8+ T cells alone. However, when anti-Bv8 antibodies were added to the MDSC and T cell co-culture, activated and effector CD8+ T cells were restored to control levels, and the levels of naïve and proliferating CD8+ T cells were significantly and substantially increased. These effects were accompanied by increased granzyme B expression measured in the cultured medium (**Figure 4C**). To further investigate the effect of MDSCs and anti-Bv8 antibodies on the functional activity of T cells, EMT6 cells were co-cultured with MDSCs and T cells in the presence or absence of anti-Bv8 antibodies, and T cell-mediated tumor cell killing was assessed by flow cytometry and Incucyte. As expected, MDSCs inhibited tumor cell killing activity of CD8+ T cells owing to their immunosuppressive function. Importantly, the addition of anti-Bv8 antibodies to the system counteracted MDSC-mediated immunosuppression, as evidenced by enhanced tumor cell killing activity (**Figures 4D, E**). These findings demonstrate that Bv8 blockade enhances the anti-tumor activity of CD8+ T cells in part by inhibiting the immunosuppressive function of MDSCs.

### MDSCs Secrete Factors Associated With Anti-Tumor Immunity in Response to Bv8 Blockade

**Figure 5:**
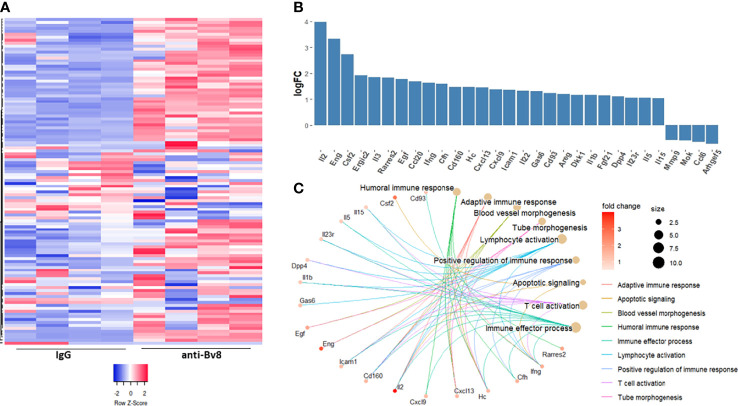
Anti-Bv8-treated MDSCs secrete proteins associated with anti-tumor immunity. MDSCs isolated form the spleen of EMT6 tumor-bearing mice were cultured with serum-free medium in the presence of anti-Bv8 or IgG control antibodies for 24 hours. Conditioned medium (CM) was applied on a murine protein array to quantify the levels of ~110 proteins as assessed by densitometry. **(A)** A heatmap representing the total proteins measured in the arrays. **(B)** The relative levels of selected proteins, plotted as a Log fold change (anti-Bv8 *vs* IgG treatment). **(C)** Selected proteins (log2FC>0.5) representing immunological biological processes were visualized by R package clusterProfiler. The size of the bubble represents the number of proteins involved in each biological pathway and the color of the bubble represents the fold change in the protein expression level.

Thus far, our findings demonstrate that Bv8 blockade inhibits the immunosuppressive function of MDSCs, thereby enhancing anti-tumor activity of cytotoxic T cells and sensitizing anti-PD1 resistant tumors. We next sought to identify the specific factors secreted by MDSCs in response to Bv8 blockade. To this end, MDSCs were isolated from the spleens of EMT6 tumor-bearing mice using magnetic beads. The cells were then cultured in the presence of anti-Bv8 or IgG control antibodies for 24 hours. Conditioned medium (CM) was collected and subsequently applied to a protein array to quantify the levels of ~110 proteins, the majority of which are immune-related factors (**Figure 5A**). Approximately 50 factors were found to be substantially elevated (Log_2_ FC>0.5) in the CM of MDSCs cultured in the presence of anti-Bv8 antibodies in comparison to the control (**Figure 5B**). The factors were associated with biological pathways such as activation of T cells, and lymphocyte activation (**Figure 5C**), in line with the *in vitro* results demonstrating enhanced cytotoxic T cell activity (**Figure 4**). Of note, some biological processes identified were associated with angiogenesis process, indicating the role of Bv8 in angiogenesis (29). Collectively, our findings demonstrate that Bv8 blockade affects MDSC function and not only recruitment to anti-PD1-treated tumors, thereby promoting biological processes associated with anti-tumor immunity.

## Discussion

Immunotherapy based on ICIs has made a paradigm shift in oncology, significantly extending response duration in cancer patients with advanced metastatic disease (30). However, the majority of patients do not respond to such therapy for reasons that are not fully understood (31). Lack of cytotoxic T cell infiltration in tumors, loss of antigen processing (32), and inadequate cytotoxic anti-tumor cell function (33) are some of the mechanisms of resistance to ICI therapy. While cytotoxic T cell-mediated adaptive immunity acts to eliminate tumor cells, innate immune cells, in particular MDSCs, promote immunosuppressive functions that impair cytotoxic T cell activity and immunosurveillance, thus supporting tumor progression (34–36). For example, one study demonstrated that MDSCs induce downregulation of L-selectin in T cells, which further inhibits T cell activity and homing to lymph nodes or tumors (37). Here, we show that orthotopically implanted breast carcinoma tumors displaying spontaneous resistance to anti-PD1 therapy exhibit significantly higher levels of MDSCs, in comparison to tumors that respond to therapy. Our findings are consistent with clinical studies reporting an increased level of MDSCs in the blood of non-responding ICI-treated melanoma patients (38, 39). Although we did not find a significant change in the peripheral blood levels of MDSC subsets, a trend towards increased levels was observed. It is possible that this trend would be significant with larger sample size. We should note that while some studies evaluate MDSC immunosuppressive function by measuring the expression of Arg1, iNOS, TGFβ, and IL-10 (as reviewed in (11)), our study primarily assessed the phenotypic characteristics of MDSC subtypes, solely based on surface markers measured by flow cytometry. Yet, we showed that the expression level of Arg1, which is known for its immunosuppressive role in myeloid cells, did not change in response to anti-Bv8 therapy, the expression of IDO, ROS1, and iNOS was substantially higher following anti-Bv8 therapy, indicating that anti-Bv8 inhibits the immunosuppressive activity of MDSCs. Overall, our findings suggest that MDSCs play a key role in counteracting the therapeutic effect of anti-PD1 treatment.

Several strategies have been proposed to overcome resistance to ICI therapy, many of which are based on combination therapies. For example, in the clinic, ICI therapy has been evaluated in combination with chemotherapy, radiation, antiangiogenic drugs and targeted therapies (40). Preclinical studies have evaluated ICI therapy in combination with drugs that inhibit immunosuppressive cells in tumors. For example, it has been shown that blocking CXCR1/2, which inhibits MDSC function and trafficking, enhances T cell activation and antitumor immunity, thereby increasing response to anti-PD1 therapy (34). Other studies demonstrated the potential inhibition of MDSC immunosuppressive activity in combination with immunotherapy to improve outcomes (41–43). In our study, we utilize anti-Bv8 antibodies to inhibit the tumor recruitment and functioning of MDSCs. We demonstrate that treating tumor-bearing mice with a combination of anti-PD1 and anti-Bv8 antibodies sensitizes tumors that are resistant to anti-PD1 monotherapy. In addition, we found higher levels of MDSCs in hyperprogressive, anti-PD1-treated tumors. The combined treatment with anti-Bv8 and anti-PD1 antibodies reduces the number of MDSCs in tumors and attenuates hyperprogression. Overall, our findings highlight the role of MDSCs in resistance to anti-PD1 therapy. Importantly, we demonstrate that Bv8 blockade sensitizes anti-PD1 resistant tumors by inhibiting, in part, the tumor colonization of MDSCs and by increasing anti-tumor immunity. It should be noted, however, that our study is limited to the analysis of MDSCs and their major subsets. It is plausible that anti-Bv8 may directly or indirectly affect other immune cell types including NK cells, B cells, T regulatory cells, and dendritic cells. The analysis of the entire immune cell composition following anti-Bv8 therapy is worthy in subsequent studies.

Bv8 blockade was initially developed as a therapeutic strategy to sensitize tumors that are refractory to antiangiogenic therapy (26). Bv8 is a secreted protein initially identified in the skin (44). Along with EG-VEGF receptor, Bv8 receptor is expressed by several types of endothelial cells, playing a key role in angiogenesis (45). Previous studies demonstrated that Bv8 regulates myeloid cell activity and supports hematopoietic cell mobilization (15). Bv8 binds to its receptor PKR2 expressed by myeloid cells. The signaling of PKR2 is through ERK phosphorylation which supports myeloid cell mobilization and chemotaxis (46). Furthermore, it has been shown that G-CSF and GM-CSF increase the expression of PKR2 expressed by immune cells further supporting myeloid cell mobilization (47). Thus, treatment with anti-Bv8 antibodies reduces the number of MDSCs both in peripheral blood and tumors, and therefore contributes to an antiangiogenic effect, and reduced tumor growth (15). Our previous studies demonstrate that combining anti-Bv8 antibodies with chemotherapy reduces MDSC colonization in treated tumors, and inhibits angiogenesis (48). Thus, the antiangiogenic effect of Bv8 blockade is related, in part, to the inhibition of MDSC trafficking. Due to increased MDSC colonization in anti-PD1-resistant tumors, we reasoned that blocking Bv8 will enhance the therapeutic activity of anti-PD1 therapy by inhibiting the trafficking of MDSCs to tumors. Indeed, we show that Bv8 blockade sensitizes spontaneously resistant EMT6 tumors as well as other intrinsically resistant tumors (i.e., LLC and RENCA models). We should note that anti-Bv8 monotherapy also induced anti-tumor activity in some of the tumor models possibly due to its antiangiogenic activity. However, this issue requires additional investigation.

Bv8 blockade is primarily known for its inhibitory effect on MDSC trafficking to tumors leading to antiangiogenic activity (48). Our study suggests that Bv8 also affects the immunosuppressive function of MDSCs. Specifically, using *in vitro* co-culture systems of MDSCs and cytotoxic CD8+ T cells, we demonstrate that cytotoxic CD8+ T cell activation is enhanced in the presence of anti-Bv8 antibodies. These results suggest that Bv8 blockade inhibits the immunosuppressive function of MDSCs, and not only their recruitment to tumors. Indeed, we show that MDSCs cultured in the presence of anti-Bv8 antibodies secrete factors associated with increased lymphocyte activity, T cell effector and anti-tumor T cell activity.

In summary, our study demonstrates the therapeutic activity of anti-Bv8 antibodies when used in combination with anti-PD1 therapy. Treatment with anti-Bv8 antibodies directly inhibits MDSC trafficking to tumors, and reduces MDSC immunosuppressive function. These effects enhance anti-tumor immunity, sensitizing anti-PD1 resistant tumors. Therefore, combining Bv8 blockade with ICI therapy represents a potential strategy for overcoming immunotherapy resistance.

## Materials and Methods

### Cell Lines

EMT6 murine breast carcinoma, LLC murine Lewis lung carcinoma, and RENCA murine renal cell carcinoma cell lines were purchased from the American Type Culture Collection (ATCC, Manassas, VA, USA) and were used within 6 months of resuscitation. Cells were routinely tested to be mycoplasma-free. Cells were grown in Dulbecco’s modified Eagle’s medium (DMEM, SigmaAldrich, Rehovot, Israel), and supplemented with 5% fetal calf serum (FCS), 1% L-glutamine, 1% sodium pyruvate, and 1% Pen-Strep-Neomycin in solution, (Biological Industries, Israel). All cells were cultured in a humidified chamber in 5% CO_2_ at 37°C.

### Murine Tumor Models

Female BALB/c or C57BL/6 mice (8-10 weeks of age) were purchased from Envigo, Israel. All mice were maintained under specific pathogen-free conditions in the animal facility located at the Rappaport Faculty of Medicine, Technion. All animal experiments were performed according to approved ethic guidelines.

EMT6 cells (5x10^5^/50 µL serum free medium) were orthotopically injected into the mammary fat pad of BALB/c female mice. RENCA and LLC cells (5x10^5^ cells/mouse) were subcutaneously injected into the flanks of 8–10-week-old female BALB/c and C57BL/6 mice, respectively. In all experiments, mice were randomly grouped before therapy. The number of mice per group is indicated in the figure. Tumor volume was measured twice a week with Vernier calipers and tumor volume was calculated by using the formula width^2^×length×0.5. When tumor size reached endpoint (approximately 1,000 mm^3^), the experiment was terminated, mice were sacrificed, and tumors were removed for further analysis.

### Treatment Schedules and Drug Dosing

Treatment with anti-PD1 (clone RMP1-14, BioXCell) and/or anti-Bv8 (Genentech, clone 2D3) antibodies was initiated when tumors reached a size of ~50 mm^3^, unless otherwise indicated in the figure. The antibodies were administered twice a week at a dose of 100µg/mouse for anti-PD1 and 5 mg/kg for anti-Bv8, for up to a 2-week period or otherwise indicated. Control mice were injected with 100 µg/mouse IgG isotype control (BioXCell).

### Single-Cell Suspension Preparation

Tumors were removed from mice, cut into small pieces, and transferred to gentleMACS™ C tubes (Miltenyi Biotec, Germany) containing 5 ml of RPMI medium supplemented with 20% FCS, 1% L-glutamine, 1% sodium pyruvate, and 1% Pen-Strep-Neomycin. Tumor pieces were subjected to homogenization using gentleMACS™ dissociator (Miltenyi Biotec, Germany), supplemented with 32 mg/ml dispase II (Godo Shusei Co., Ltd, Tokyo, Japan) and 38 mg/ml collagenase type 1 (Worthington Biochemical Corp, Lakewood, NJ, USA) and were incubated for 1 hour at 37°C in a shaker incubator. Tumor homogenates were strained through cell strainers (70 µl mesh size) into 50 ml tubes and subsequently centrifuged at 470 x g for 5 min. The supernatant is discarded, and the cell pellet was used for further analysis as described below. Peripheral blood (PB) was collected by submandibular vein puncture. The blood was collected into a tube containing 50 µl 0.1M EDTA. Red blood cells (RBCs) in tumor suspensions and peripheral blood were lysed using RBC-lysis buffer (0.8% ammonium chloride, Alfa Aesar, Haverhill, MA, USA, and 0.1M EDTA, Biological Industries, Israel). Pellets containing the isolated single cells were resuspended in PBS to the required volume for further experimental procedures and analysis.

### Flow Cytometry Acquisition and Analysis

Different immune cell types in single-cell suspensions of tumor tissue or peripheral blood were quantified by flow cytometry. Cells were immunostained for different surface markers to define immune cell types including non-activated and activated CD8+ T cells, as well as granulocytic and monocytic MDSCs, as indicated in **Table S1**. The flow cytometry gating strategy is shown in **Figure S8A**. All monoclonal antibodies were purchased from BD Biosciences, R&D systems, or Macs Militenyi Biotec, and were used in accordance with the manufacturers’ instructions. At least 300,000 events were acquired using a Fortessa flow cytometer (BD Biosciences) and analyzed by FlowJo 7.6.1. (FlowJo, Ashland, OR).

### *In Vitro* Co-Culture Assay

Gr1+ cells and CD8+ T cells were isolated from the spleens of EMT6 tumor-bearing mice using positive isolation EasySep Mouse PE, BioLegend, and MojoSort™ Mouse CD8 T Cell Isolation Kit, respectively. The Gr1+ cells (2x10^5^ cells/ml) were seeded in 24 well plates and co-cultured with anti-Bv8 or IgG control antibodies (10 µg/ml) for 24 hours. Cells were then collected and immunostained for G-MDSCs or M-MDSCs. To analyze cell viability, the cells were stained with 7AAD and propidium iodide (PI) and analyzed by flow cytometry. In some experiments, Gr1+ cells (0.5x10^6^ cells/ml) and CD8+ T cells (0.5x10^6^ cells/ml) were co-cultured in a 24-well plate in the presence of anti-Bv8 or IgG control antibodies for 24 hours. Subsequently, the cells were collected and analyzed by flow cytometry to detect different immune cell states as indicated in the figure and in **Table S1**. The flow cytometry gating strategy is shown in **Figure S8B**. In some experiments, granzyme B was evaluated in the conditioned medium obtained from the co-cultured system analyzed by ELISA (R&D systems). All experiments were performed at least in three biological replicates.

### Tumor Cell Killing Assay

Gr1+ cells and CD8+ T cells were isolated from the spleens of EMT6 tumor-bearing mice (n=5 mice) using positive isolation EasySep Mouse PE, BioLegend, and MojoSort™ Mouse CD8 T Cell Isolation Kit, respectively. EMT6 cells were seeded in a 48-well plate (4000 cells/well) along with CD8+ T cells (0.5x10^6^ cells/ml) and Gr1+ cells (0.5x10^6^ cells/ml) obtained from each individual mouse, for 24 hours in the presence of anti-Bv8 or IgG control antibodies (10 µg/ml). Subsequently, PI (500 nM) was added to cultures in order to identify dead cells. T-cell killing effect was monitored by flow cytometry and Incucyte Zoom HD/2CLR system (Essen BioScience, Ann Arbor, MI). The flow cytometry gating strategy is shown in **Figure S8C**.

### Real-Time Quantitative PCR

mRNA was extracted from cancer cells including EMT6, RENCA and LLC cell lines or from GR1+ or CD8+ T cells isolated from EMT6 tumor bearing BALB/c mice, as indicated in the text. The mRNA was extracted using total RNA purification kit (Norgen Biotek, Thorold, ON, Canada), in accordance with the manufacturer’s protocol. Complementary DNA (cDNA) was then synthesized from the mRNA samples using High-Capacity cDNA Reverse Transcription Kit (Applied Biosystems, CA). Real-time quantitative PCR (RT-qPCR) reaction was performed using SYBR Green Master Mix and run in CFX Connect Real-Time PCR Detection System (Bio-Rad Laboratories, Hercules, CA). Analysis was performed using the ΔΔC_t_ method, while Hsp90 served as a house keeping gene control. Primers are listed in **Table S2**.

### Cytokine Array and Biological Pathway Analysis

Gr1+ cells were isolated from the spleen of EMT6 tumor-bearing mice using EasySep Mouse PE. Cells were cultured in the presence of anti-Bv8 or IgG control antibodies (10 µg/ml), for 24 hours. Cells (1x10^6^/ml) were washed, and serum-free medium was then added. After 24 hours, conditioned medium (CM) was applied on a cytokine array (Proteome Profiler Mouse XL Cytokine Array, R&D systems) in accordance with the manufacturer’s instruction. Relative levels of the different proteins from 4 biological samples were calculated using DESeq2 R package v1.34 (49). Heatmap based on normalized proteins was generated using the website tool www.heatmapper.ca. Data is presented as the Log_2_ fold change (anti-Bv8 *vs* IgG control). Proteins with log_2_FC>0.5 and log_2_FC<-0.5 were selected, and further investigated for their association with relevant biological pathways. The biological pathways were selected based on Gene Ontology (GO) category *biological process* used as a reference*. The biological processes* were then visualized by R package clusterProfiler v4.2.2 (50).

### Statistical Analysis

Data were expressed as mean ± standard deviation (SD). The statistical significance of differences was assessed by one-way ANOVA, followed by Tukey *post hoc* statistical test using GraphPad Prism 5 software (La Jolla, CA). When the comparison was carried out between two groups, unpaired student t test was performed. Differences between all groups were compared with each other and were considered significant at p values below 0.05.

## Data Availability Statement

The original contributions presented in the study are included in the article/**Supplementary Material**. Further inquiries can be directed to the corresponding author.

## Ethics Statement

The animal study was reviewed and approved by the Technion's Animal Ethic committee.

## Author Contributions

Conception and design: MB and YS. Acquisition of data: MB, AV, SL, MT, JH-S, AD, TC, RM, and ZR. Analysis and interpretation of data: MB, SL, TC, ZR, and YS. Writing, review, and/or revision of the manuscript: MB and YS. Study supervision: YS. All authors contributed to the article and approved the submitted version.

## Funding

This work is supported primarily by H2020 European Research Council Grant (771112), Israel Science Foundation (194/18), and Nikoh foundation awarded to YS. MB and JH-S are supported by Ariane de Rothschild Women Doctoral Program. TC is supported by RTICC-Rubinstein fellowship.

## Conflict of Interest

The authors declare that the research was conducted in the absence of any commercial or financial relationships that could be construed as a potential conflict of interest.

## Publisher’s Note

All claims expressed in this article are solely those of the authors and do not necessarily represent those of their affiliated organizations, or those of the publisher, the editors and the reviewers. Any product that may be evaluated in this article, or claim that may be made by its manufacturer, is not guaranteed or endorsed by the publisher.
