# Supplementary material for: Bv8 Blockade Sensitizes Anti-PD1 Therapy Resistant Tumors
Source: Front Immunol. 2022 Jul 7;13:903591. doi: 10.3389/fimmu.2022.903591 (PMC9301046; doi:10.3389/fimmu.2022.903591)
Supplement: Supplementary file 1 [file DataSheet_1.pdf]

Supplemental tables and Figures

Benguigui et. al,

Supplemental tables

Table S1: The definition of selected immune cells by surface markers

**Cell type**	**Surface markers**
G-MDSC	$CD45^{+}CD11B^{+}LY6C^{LOW}LY6G^{+}$
M-MDSC	$CD45^{+}CD11B^{+}LY6C^{+}LY6G^{-}$
Non-activated $CD8^{+}$ T cell	$CD45^{+}CD8^{+}$ $CD25^{-}$
Activated $CD8^{+}$ T cell	$CD45^{+}CD8^{+}$ $CD25^{+}$
Effector/Memory $CD8^{+}$ T cell	$CD45^{+}CD8^{+}$ $CD44^{+}$ $CD62L^{-}$
Naïve $CD8^{+}$ T cell	$CD45^{+}CD8^{+}$ $CD44^{-}$ $CD62L^{+}$
Proliferating $CD8^{+}$ T cell	$CD45^{+}CD8^{+}$ $Ki67^{+}$

Table S2: List of primers used for RT-PCR.

**Gene**	**Forward**	**Reverse**
PKR2	TTTGTGCCTCCGTCAACTACC	TTCAAAGGGTGGACAATAGCG
Bv8	GCCCCGCTACTGCTACTTC	CCGCACTGAGAGTCCTTGTC
IDO	GCTTTGCTCTACCACATCCAC	CAGGCGCTGTAACCTGTGTC
ROS1	GCTGCCTAACGTCCTGTGTAA	GGTGCTGTAGGAAGTTCTGTG
iNOS	ACATCGACCCGTCCACAGTAT	CAGAGGGGTAGGCTTGTCTC
Arg1	CTCCAAGCCAAAGTCCTTAGAG	GGAGCTGTCATTAGGGACATCA
Hsp90	TCGTCAGAGCTGATGATGAAGT	GCGTTTAACCCATCCAACTGAAT

Supplemental Figures

Figure S1

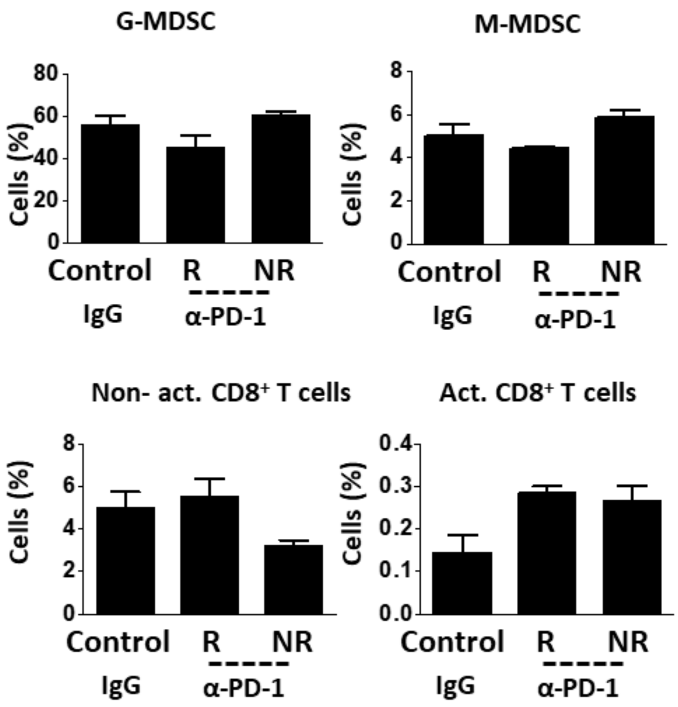

Figure S1: Quantification of MDSCs and T cells in peripheral blood of mice displaying response and resistance to anti-PD1 therapy. Peripheral blood was obtained from EMT6 tumor-bearing mice at the endpoint after treatment with IgG control or anti-PD1 responder (R) and non-responder (NR) mice, as described in Figure 1 (n=5-6 mice/group). Granulocytic and monocytic MDSCs (G-MDSC and M-MDSC, respectively) as well as non-activated and activated CD8+ T cells were analyzed using flow cytometry. The results are presented as the percentage from CD45+ cells. The average percentage ± SD for each cell type, is shown in a bar graph. Statistical significance was assessed by one-way ANOVA followed by Tukey post-hoc test. No significant p values were found.

Figure S2

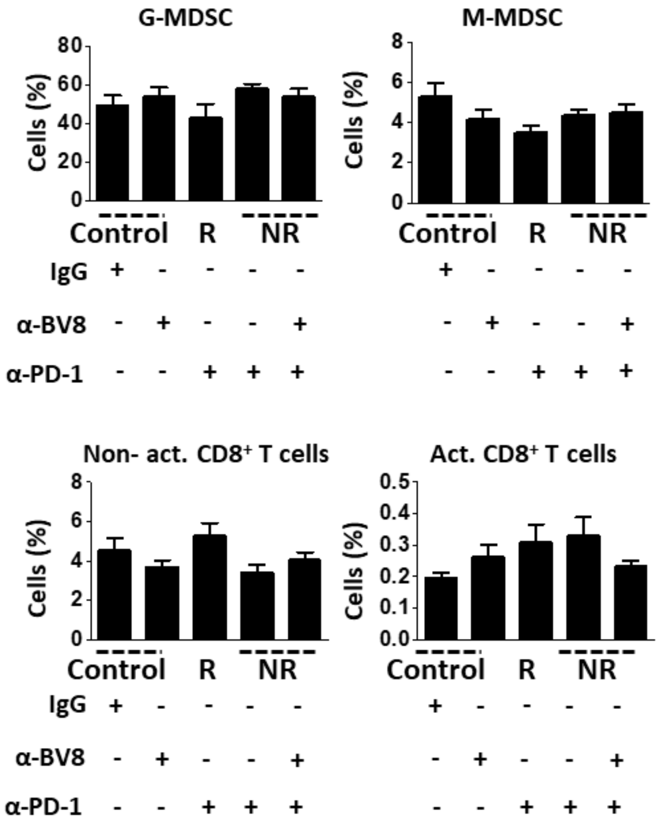

Figure S2: Anti-Bv8 treatment inhibits the enrichment of MDSCs in anti-PD1 resistant tumors. Peripheral blood was obtained from EMT6 tumor-bearing mice at the endpoint after treatment with IgG control, anti-Bv8 and/or anti-PD1, as described in Figure 2 (n=6-10 mice/group). Granulocytic and monocytic MDSCs (G-MDSC and M-MDSC, respectively) as well as non-activated and activated CD8+ T cells were analyzed using flow cytometry. The results are presented as the percentage from CD45+ cells. The average percentage ± SD for each cell type is shown in a bar graph. Statistical significance was assessed by one-way ANOVA followed by Tukey post-hoc test. No significant p values were found.

Figure S3

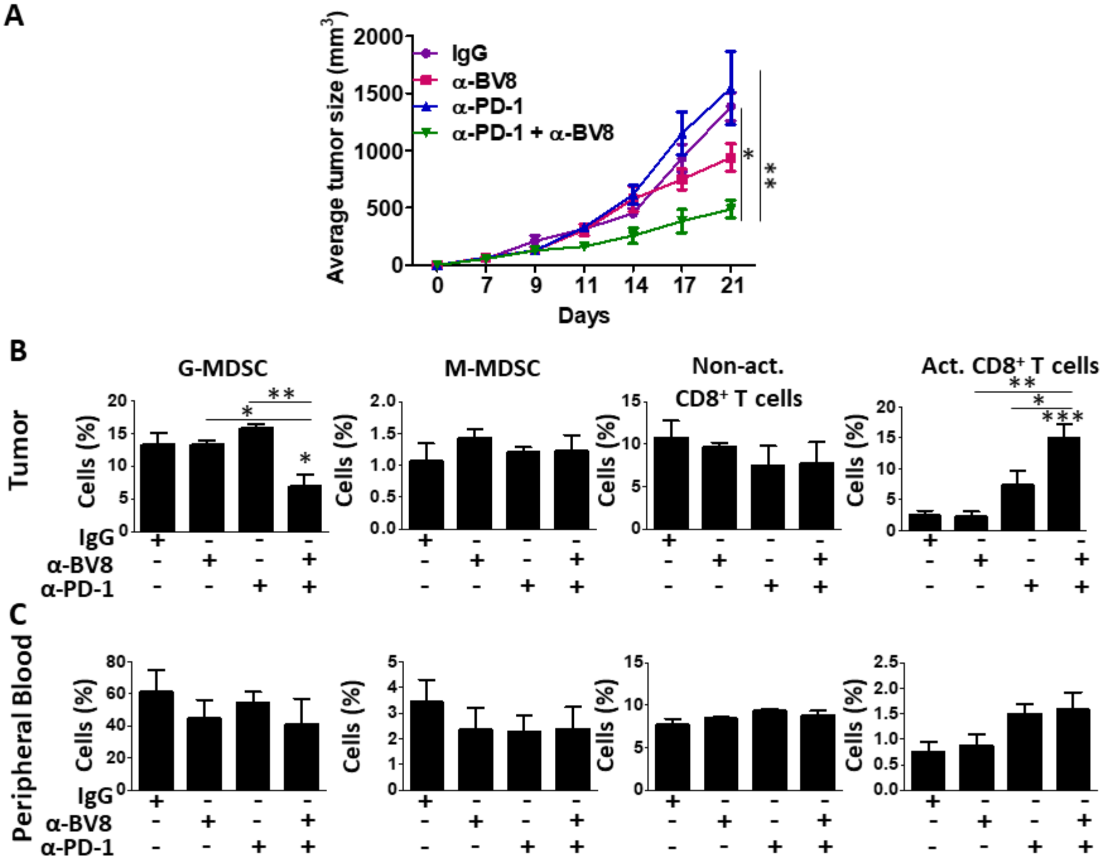

Figure S3: Anti-Bv8 treatment sensitizes LLC tumors otherwise resistant to anti-PD1 therapy. Eight-to-ten week old C57BL/6 mice (n=5 mice/group) were subcutaneously implanted with LLC cells ($5x10^5$ cells/mouse). When tumors reached $50mm^3$, treatment with anti-PD1 and/or anti-Bv8 antibodies was initiated twice weekly. Control mice were treated with IgG control. (A) Tumor growth was assessed regularly. (B-C) At the endpoint, tumors and peripheral blood were harvested. Granulocytic and monocytic MDSCs (G-MDSC and M-MDSC, respectively) in tumor single-cell suspensions (B) and peripheral blood (C) were quantified by flow cytometry. The results are presented as the percentage from CD45+ cells. The average percentage ± SD for each cell type, is shown in a bar graph. Statistical significance was assessed by one-way ANOVA followed by Tukey post-hoc test. Significant p values are shown as * $p<0.05$; ** $p<0.01$ from control or otherwise indicated in the figure.

Figure S4

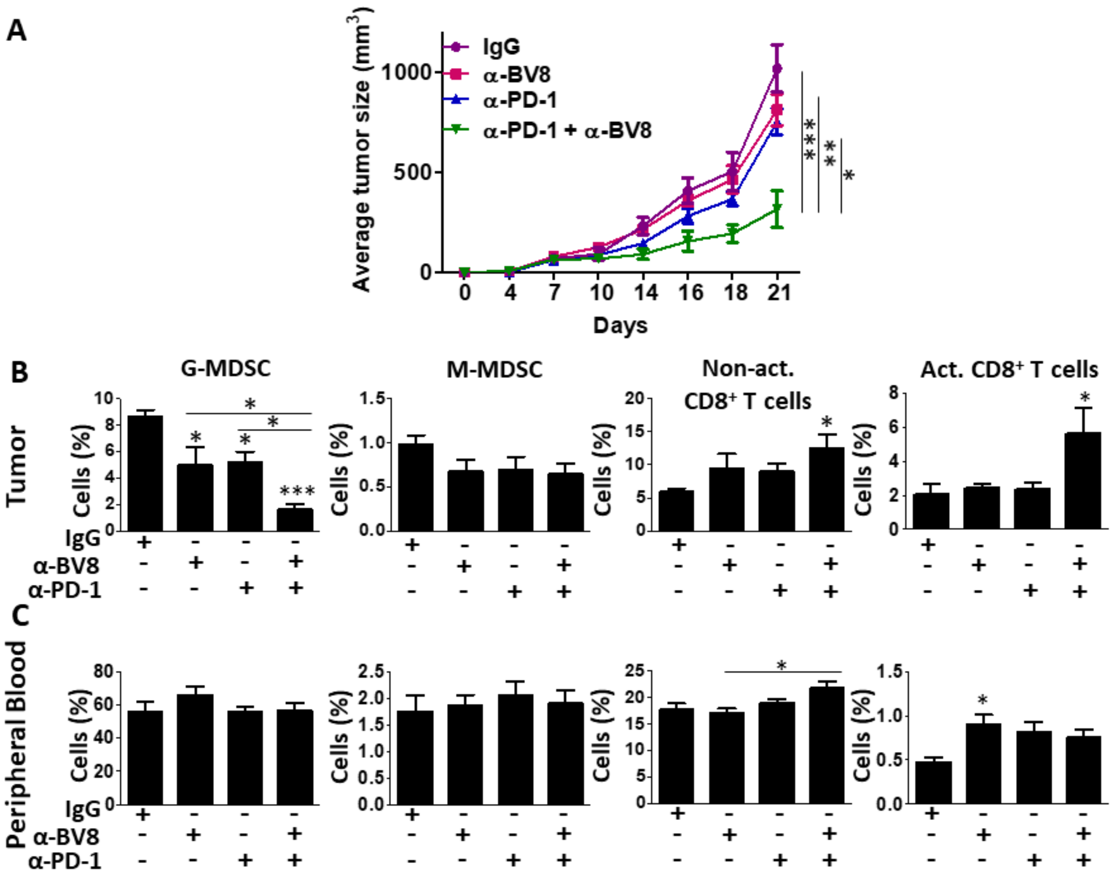

Figure S4: Anti-Bv8 treatment sensitizes RENCA tumors otherwise resistant to anti-PD1 therapy. Eight-to-ten week old BALB/c mice (n=5 mice/group) were subcutaneously implanted with RENCA cells ($5x10^5$ cells/mouse). When tumors reached $50mm^3$, treatment with anti-PD1 and/or anti-Bv8 antibodies was initiated twice weekly. Control mice were treated with IgG control. (A) Tumor growth was assessed regularly. (B-C) At the endpoint, tumors and peripheral blood were harvested. Granulocytic and monocytic MDSCs (G-MDSC and M-MDSC, respectively) in tumor single-cell suspensions (B) and peripheral blood (C) were quantified by flow cytometry. The results are presented as the percentage from CD45+ cells. The average percentage ± SD for each cell type, is shown in a bar graph. Statistical significance was assessed by one-way ANOVA followed by Tukey post-hoc test. Significant p values are shown as * p<0.05; ** p<0.01; *** p<0.001 from control or otherwise indicated in the figure.

Figure S5

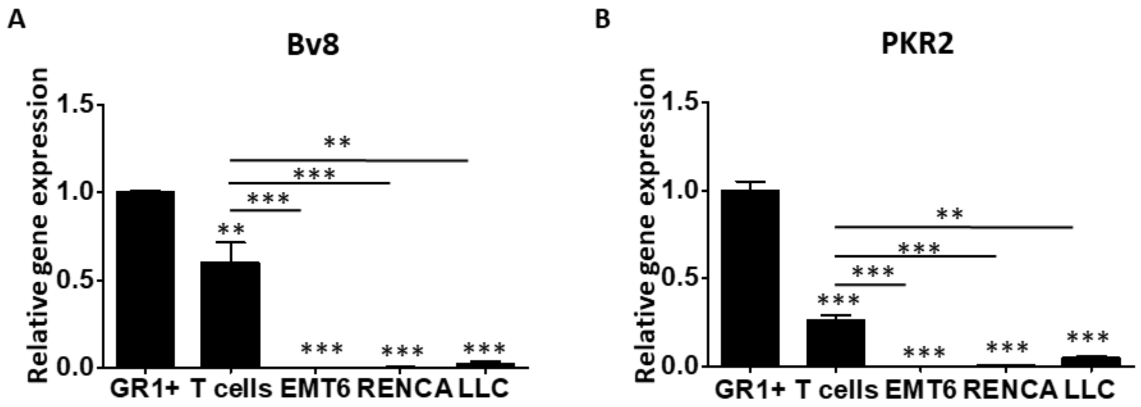

Figure S5: The relative mRNA expression of Bv8 and PKR2 in different cell types. (A-B)Gr1+ and T cells isolated from tumors, as well as EMT6, RENCA, and LLC cell lines were prepared for mRNA extraction. mRNA levels were analyzed by RT-PCR for Bv8 (A) and PKR2 (B) mRNA expression. The relative mRNA expression is shown in a bar graph. Significance was assessed by one-way ANOVA followed by Tukey post-hoc test. Significant p values are shown as ** $p<0.01$; *** $p<0.001$ from control or otherwise indicated in the figure.

Figure S6

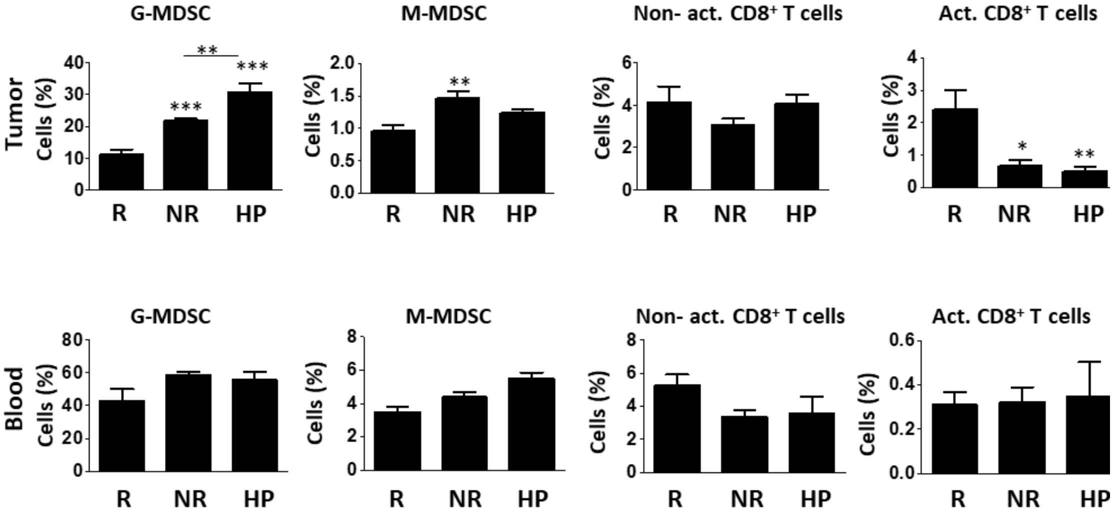

Figure S6: The composition of MDSCs and T cells in mice bearing EMT6 hyperprogressive tumor phenotype. Tumor and peripheral blood were obtained from EMT6 tumor-bearing mice at the endpoint (day 17) after treatment with anti-PD1, when the mice were stratified to responders (R), non-responders (NR) and hyperprogressive tumor phenotype (HR), similar to that described in Figure 3 (n=5-8 mice/group). Granulocytic and monocytic MDSCs (G-MDSC and M-MDSC, respectively) as well as non-activated and activated CD8+ T cells were analyzed using flow cytometry. Results are presented as the percentage from CD45+ cells. The average percentage ± SD for each cell type is shown in a bar graph. Statistical significance was assessed by one-way ANOVA followed by Tukey post-hoc test. Significant p values are shown as * $p<0.05$; ** $p<0.01$; *** $p<0.001$ from control or otherwise indicated in the figure.

Figure S7

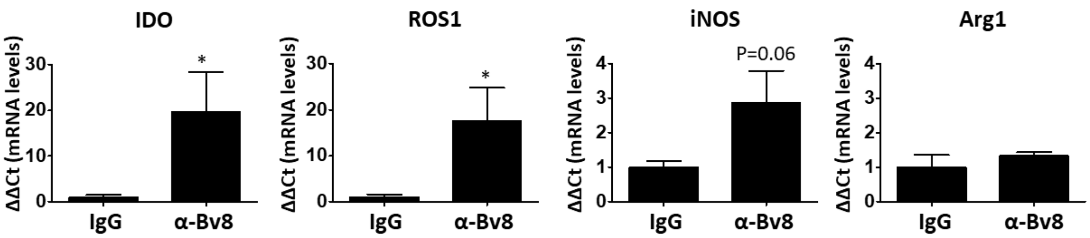

Figure S7: mRNA levels of immunomodulating genes in MDSCs from anti-Bv8 or control EMT6 tumor-bearing mice. EMT6 tumor-bearing mice were treated with anti-Bv8 or IgG control. After one week of treatment (3 injections), Gr1+ cells were isolated from the tumors, and mRNA was extracted. The mRNA levels of IDO, ROS1, iNOS, and Arg1 were assessed and presented as relative to IgG control. Statistical significance was assessed using an unpaired two-tailed t-test. Significant p values are shown as * $p<0.05$.

Figure S8

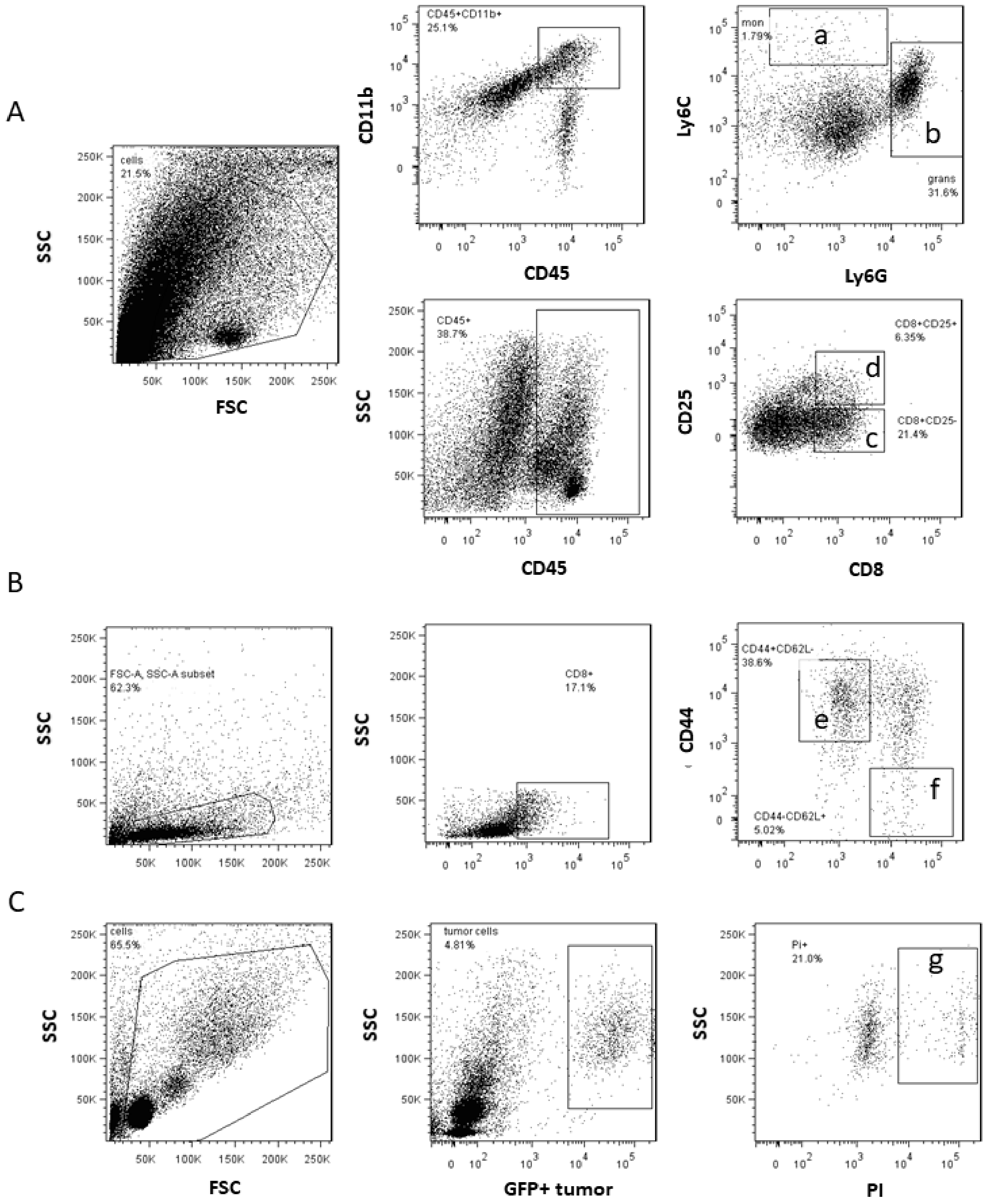
A
B
C
SSC
FSC
cells
21.5%
CD11b
CD45
CD45+CD11b+
25.1%
Ly6C
Ly6G
mon
1.79%
grans
31.6%
a
b
CD45+
38.7%
CD25
CD8
CD8+CD25+
6.35%
CD8+CD25-
21.4%
d
c
FSC-A, SSC-A subset
62.3%
CD8+
17.1%
CD44
CD44+CD62L-
38.6%
CD44-CD62L+
5.02%
e
f
cells
65.5%
tumor cells
4.81%
GFP+ tumor
PI+
21.0%
PI
g

Figure S8: Flow cytometry gating strategy. Flow cytometry analysis of the different cell types is shown by representative dotplots. (A) Tumor single-cell suspension gating strategy of CD11b+ monocytic MDSCs (a, Ly6C+/Ly6G-)) and granulocytic MDSCs (b. Ly6C-/Ly6G+) or of non-activated CD8+ T cells (c, CD8+/CD25-) and activated CD8+ T cells (d, CD8+/CD25+). Peripheral blood samples were analyzed the same way. (B) The gating strategy of effector/memory CD8+ T cells (e, CD44+/CD62L-) or naive T cells (f, CD44-/CD62L+). (C) The gating strategy of dead tumor cells (g, tumor cell-GFP+/PI+).
